# Supplementary material for: The origin of the Moon’s Earth-like tungsten isotopic composition from dynamical and geochemical modeling
Source: Nat Commun. 2021 Jan 4;12:35. doi: 10.1038/s41467-020-20266-1 (PMC7782809; doi:10.1038/s41467-020-20266-1)
Supplement: Supplementary file 1 — Supplementary Information [file 41467_2020_20266_MOESM1_ESM.pdf]

Supplementary Information for:

The origin of the Moon's Earth-like tungsten isotopic composition from dynamical and geochemical modeling

Rebecca A. Fischer, Nicholas G. Zube, and Francis Nimmo

This file contains:

Supplementary Figures 1 and 2

Supplementary Figures

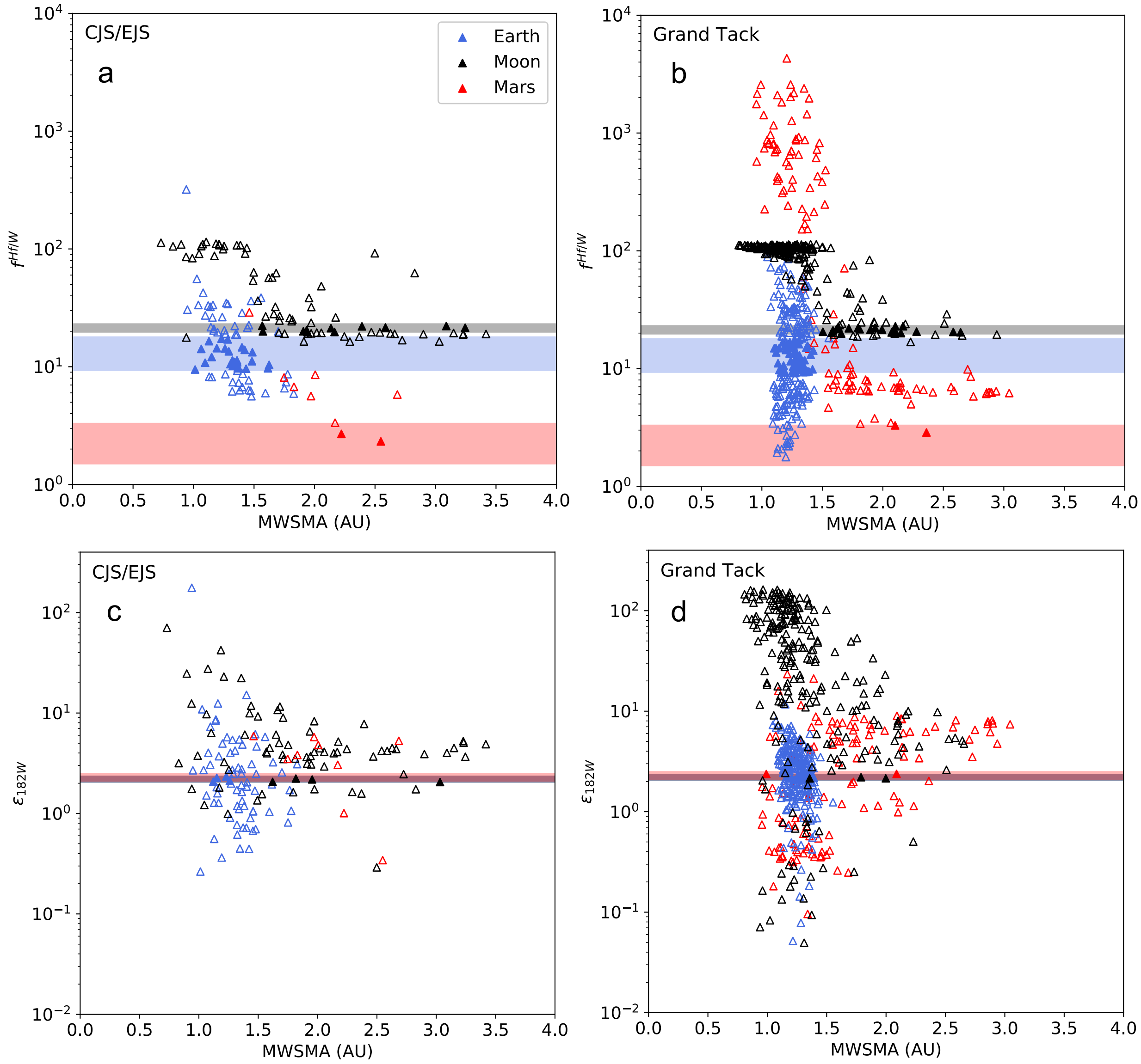

Supplementary Figure 1 | $f^{\mathrm{Hf/W}}$ (a and b) and ε_{182W} (c and d) of final Earth, Moon, and Mars analogues with different mass-weighted semimajor axes (MWSMA). Results are shown for Circular Jupiter and Saturn (CJS) and Eccentric Jupiter and Saturn (EJS) accretion simulations, combined (a and c), or Grand Tack simulations (b and d). The degree of metal equilibration was *k* = 0.4 for CJS/EJS calculations (ref. 1–2) and *k* = 0.8 for Grand Tack calculations (ref. 3). Whole mantle equilibration was

assumed, with a lunar W metal–silicate partition coefficient D_W = 250. Shaded regions indicate observed $f^{Hf/W}$ values (4) or observed/pre-late veneer ε_{182W} values (5–6; Shergottite group value shown for Mars). Open symbols: all Earth, Moon, and Mars analogues. Filled symbols: analogues that match the observed $f^{Hf/W}$ or ε_{182W} values. All results were generated with the full model (Methods). Earth analogues (blue) have an Earth-like $f^{Hf/W}$ and ε_{182W} for MWSMA of ~1.0–1.6 AU. Mars analogues (red) have a Mars-like $f^{Hf/W}$ for MWSMA of ~2–3 AU. Moon analogues (black) have a Moon-like $f^{Hf/W}$ and ε_{182W} for MWSMA of ~1.6–3.3 AU, more similar to Mars than to Earth. Making a Moon with the correct $f^{Hf/W}$ and ε_{182W} requires its provenance to be different from Earth's, suggesting that its stable isotopes may also be different.

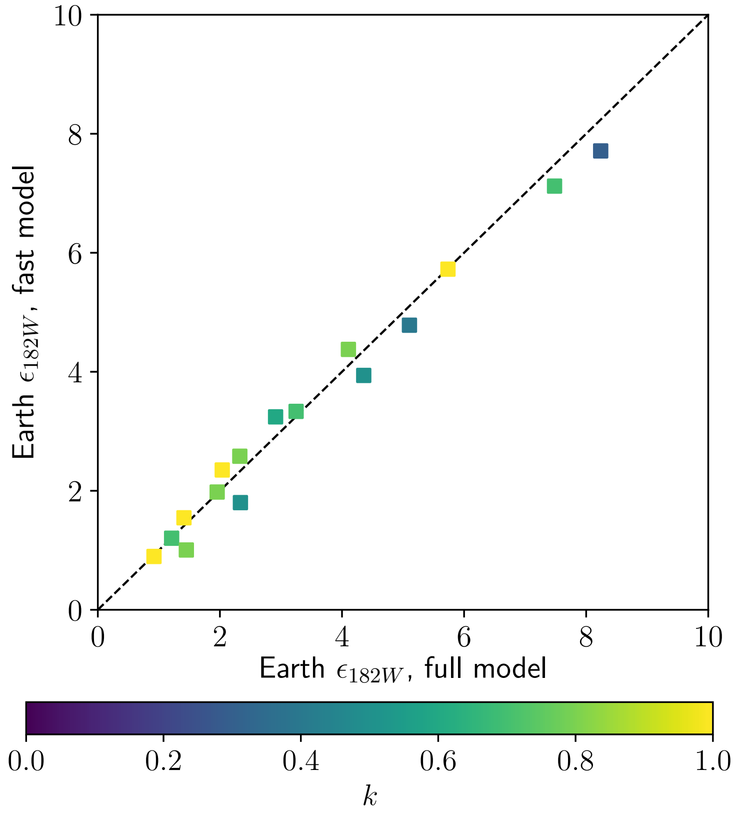

Supplementary Figure 2 | Results of benchmarking the fast model and full model of core formation on Grand Tack *N*-body simulations. Calculations are shown for 8 Earth analogues from 4 simulations (ref. 7), for variable degrees of metal equilibration *k* (indicated by color). Black dashed line is a 1:1 line.

Supplementary References

1. Nimmo, F., O'Brien, D. P. & Kleine, T. Tungsten isotopic evolution during late-stage accretion: Constraints on Earth–Moon equilibration. *Earth Planet. Sci. Lett.* **292,** 363–370 (2010).
2. Fischer, R. A. & Nimmo, F. Effects of core formation on the Hf–W isotopic composition of the Earth and dating of the Moon-forming impact. *Earth Planet. Sci. Lett.* **499,** 257–265 (2018).
3. Zube, N. G., Nimmo, F., Fischer, R. A. & Jacobson, S. A. Constraints on terrestrial planet formation timescales and equilibration processes in the Grand Tack scenario from Hf–W isotopic evolution. *Earth Planet. Sci. Lett.* **522,** 210–218 (2019).
4. Kleine, T. et al. Hf–W chronology of the accretion and early evolution of asteroids and terrestrial planets. *Geochim. Cosmochim. Acta* **73,** 5150–5188 (2009).
5. Kruijer, T. S., Kleine, T., Fischer-Gödde, M. & Sprung, P. Lunar tungsten isotopic evidence for the late veneer. *Nature* **520,** 534–537 (2015).
6. Kleine, T., Mezger, K., Münker, C., Palme, H. & Bischoff, A. ^{182}Hf–^{182}W isotope systematics of chondrites, eucrites, and martian meteorites: Chronology of core formation and early mantle differentiation in Vesta and Mars. *Geochim. Cosmochim. Acta* **68**, 2935–2946 (2004).
7. Jacobson, S. A. & Morbidelli, A. Lunar and terrestrial planet formation in the Grand Tack scenario. *Phil. Trans. R. Soc. A* **372,** 20130174 (2014).
