# Supplementary material for: The origin of the Moon’s Earth-like tungsten isotopic composition from dynamical and geochemical modeling
Source: Nat Commun. 2021 Jan 4;12:35. doi: 10.1038/s41467-020-20266-1 (PMC7782809; doi:10.1038/s41467-020-20266-1)
Supplement: Supplementary file 3 — Description of Additional Supplementary Files [file 41467_2020_20266_MOESM3_ESM.pdf]

Description of Additional Supplementary Files

File Name: Supplementary Data 1

Description: Terrestrial and lunar e182W and f Hf/W. Results are shown for accretion simulations run under the Eccentric Jupiter and Saturn (EJS) (24), Circular Jupiter and Saturn (CJS) (24), and Grand Tack (25) scenarios, and for various degrees of metal equilibration (k). All calculations use whole mantle equilibration and a lunar W metal–silicate partition coefficient DW = 30. CJS/EJS calculations were performed using the full model, and Grand Tack calculations were performed using the fast model (Methods).
